# Pharmacological Blockade of the Adenosine A_2B_ Receptor Is Protective of Proteinuria in Diabetic Rats, through Affecting Focal Adhesion Kinase Activation and the Adhesion Dynamics of Podocytes

**DOI:** 10.3390/cells13100846

**Published:** 2024-05-16

**Authors:** Pablo Mendoza-Soto, Claudia Jara, Ángelo Torres-Arévalo, Carlos Oyarzún, Gonzalo A. Mardones, Claudia Quezada-Monrás, Rody San Martín

**Affiliations:** 1 Molecular Pathology Laboratory, Institute of Biochemistry and Microbiology, Science Faculty, Universidad Austral de Chile, Valdivia 5110566, Chile; pa.men2a@gmail.com (P.M.-S.); claudia.jaracancino@gmail.com (C.J.); atorres32@santotomas.cl (Á.T.-A.); carlosoyarzun@uach.cl (C.O.); 2 Institute of Physiology, Medicine Faculty, Universidad Austral de Chile, Valdivia 5090000, Chile; gonzalo.mardonesc@uss.cl; 3 Tumor Biology Laboratory, Institute of Biochemistry and Microbiology, Science Faculty, Universidad Austral de Chile, Valdivia 5110566, Chile; claudiaquezada@uach.cl; 4 Millennium Institute on Immunology and Immunotherapy, Universidad Austral de Chile, Valdivia 5110566, Chile

**Keywords:** diabetic nephropathy, proteinuria, podocyte effacement, adenosine receptors

## Abstract

Induction of the adenosine receptor A_2B_ (A_2B_AR) expression in diabetic glomeruli correlates with an increased abundance of its endogenous ligand adenosine and the progression of kidney dysfunction. Remarkably, A_2B_AR antagonism protects from proteinuria in experimental diabetic nephropathy. We found that A_2B_AR antagonism preserves the arrangement of podocytes on the glomerular filtration barrier, reduces diabetes-induced focal adhesion kinase (FAK) activation, and attenuates podocyte foot processes effacement. In spreading assays using human podocytes in vitro, adenosine enhanced the rate of cell body expansion on laminin-coated glass and promoted peripheral pY397-FAK subcellular distribution, while selective A_2B_AR antagonism impeded these effects and attenuated the migratory capability of podocytes. Increased phosphorylation of the Myosin2A light chain accompanied the effects of adenosine. Furthermore, when the A_2B_AR was stimulated, the cells expanded more broadly and more staining of pS19 myosin was detected which co-localized with actin cables, suggesting increased contractility potential in cells planted onto a matrix with a stiffness similar to of the glomerular basement membrane. We conclude that A_2B_AR is involved in adhesion dynamics and contractile actin bundle formation, leading to podocyte foot processes effacement. The antagonism of this receptor may be an alternative to the intervention of glomerular barrier deterioration and proteinuria in the diabetic kidney disease.

## 1. Introduction

Chronic kidney disease (CKD) is an irreversible condition that progressively reduces kidney function [1,2]. The progression of CKD is characterized by proteinuria, accumulative damage to the nephrons, and the subsequent atrophy of the reabsorbing tubules. The greater contribution to this health catastrophe comes from diabetes [3,4], affecting more than half a billion people worldwide [5], with 40% of them evolving to diabetic nephropathy (DN) [6] because of metabolic, hemodynamic, and inflammatory alterations that occur in the diabetic kidney [7]. At the terminal stage, patient survival is dependent on expensive organ replacement therapies such as hemodialysis and kidney transplants [2]. This disease disproportionally affects disadvantaged populations and incapacitates millions every year while at the same time demanding significant resources from public health systems [8], without improving the odds of remission nor the patient’s quality of life [9].

The basis of proteinuria in diabetic CKD relies on alterations at the glomerular filtration barrier (GFB). The GFB is a 3-layered filtering sieve resulting from the enwrapment of a fenestrated endothelium with a 300-nanometer thick basement membrane and specialized epithelia of octopus-shaped podocytes enwrapping the urinary face of the barrier [10]. The podocytes interlock their thick actin cytoskeleton membrane projections, denominated foot processes (FPs), with their adjacent neighbors in a Turing pattern [11], which is a key arrangement for the filtration process. FPs disposition depends on two specialized junctional nodes, the slit-diaphragm and the integrin-based adhesome, each one constituted of distinct components and functionally different communication with the intracellular cytoskeleton. The slit diaphragm multiprotein complexes link the FPs of neighboring podocytes, contributing to the filtration mesh, while integrin-based adhesions anchor FPs to the basement membrane and dynamically counteract filtration flow stress through acto-myosin contractility [12]. Podocytes may undergo tremendous changes in shape in response to the diabetic milieu, essentially modifying the strength of their attachment over the basement membrane by remodeling the architecture of their FPs and their associated adhesome [13]. This adaptation, known as foot processes effacement (FPE), is observable in early onset DN, which leads to the simplification of the pedicellar phenotype of podocytes and results in a leakage of molecules normally retained in the plasma and decreases the ability of podocytes to attach to and compress the basal membrane, finally leading to podocyte detachment [14]. Some cellular components involved in the promotion of improper podocyte motility are strongly related to FPE. The downregulation of SRGAP2a, which suppresses podocyte motility by inactivating RhoA and Cdc42, occurs in both DN patients and db/db mice; and increasing podocyte SRGAP2a levels ameliorates both podocyte injury and proteinuria in db/db mice [15]. The ROCK kinases, myosin light chain phosphorylating factors that promote podocyte contractility, are activated in models of diabetes both in vivo and in vitro. Thus, the inhibition of ROCK was protective against the development of DN in mice [16,17,18]. Focal adhesion kinase (FAK) is a non-receptor tyrosine kinase, in which integrin- or growth factor–induced autophosphorylation at tyrosine 397 results in the activation of critical signaling pathways required for focal adhesion turnover and cell migration. FAK activation initiates focal adhesion instability resulting in the retraction of the FPs, and thus, the deletion of FAK in podocytes resulted in diminished proteinuria and FPE after induced glomerular injury in mice [19,20]. However, the effectiveness of FAK intervention in DN has yet to be determined. Currently, there is a critical need for therapeutic alternatives to alleviate FPE and proteinuria in diabetic and non-diabetic CKD [21]. Through genetic approaches in experimental models, some critical targets have been identified; however, translation to clinically useful interventions remain elusive.

Adenosine is an endogenous nucleoside that triggers cellular effects by signaling through adenosine A_1_, A_2A_, A_2B_, and A_3_ receptor subtypes, characterized by particular signaling properties and affinities with the ligand [22]. Adenosine mediates relevant physiological actions in the normal kidney, such as the dynamic control of the glomerular filtration rate through adenosine A_1_ receptor subtype-dependent tubuloglomerular feedback [23]. Furthermore, the adenosine A_2A_ receptor takes on a renoprotective role and may preserve structure and function of podocytes in an experimental model of podocyte injury [24]. However, adenosine signaling properties seem altered in the diabetic kidney and growing evidence links DN occurrence with the pathogenic signaling of increased adenosine [22]. A correlation was found between the increased concentration of adenosine in the plasma or urine and DN development in diabetic people [25,26]. In animal models, increased adenosine levels parallel with morphological and functional alterations that resemble incipient DN [27,28,29]. While the expression of the four adenosine receptor subtypes has been detected in murine glomeruli and podocytes [26], the lower affinity adenosine A_2B_ receptor subtype (A_2B_AR) is induced in the renal glomeruli during the progression of kidney damage both in the experimental model and in human DN [22,30] which matched with the increased ligand level. Interestingly, the selective antagonism of A_2B_AR in streptozotocin-induced diabetic rats attenuated the biochemical and histopathological signs of glomerulopathy, with a remarkable attenuation of the proteinuria [31]. Transcriptomic analysis of the glomeruli from diabetic rats evidenced that A_2B_AR antagonism affected gene transcripts belonging to the focal adhesion and cell adhesion gene ontology categories [31]. Thus, this study was designed to evaluate how podocytes interact with the extracellular matrix and how pathogenic adenosine signaling may affect the effacement process. Furthermore, we will identify the targets modified through A_2B_AR antagonism that may confer beneficial outcomes in the diabetic kidney disease model.

## 2. Results

### 2.1. The A_2B_AR Antagonist Attenuates Diabetes-Induced FPE In Vivo

**Figure 1:**
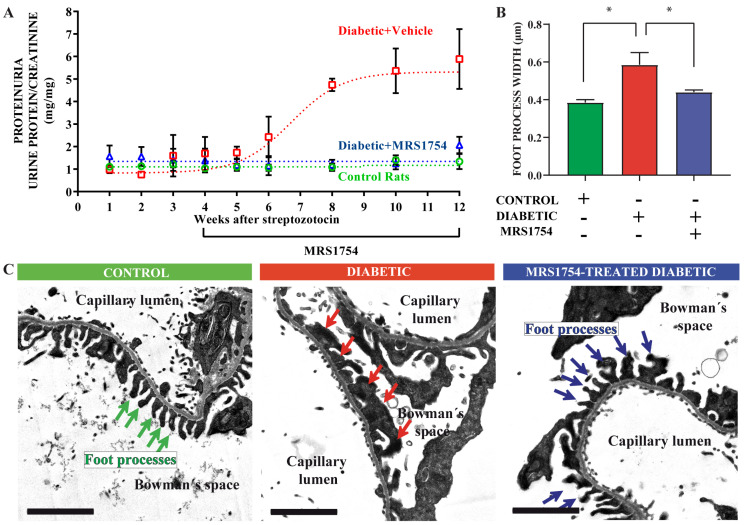
Evaluation of A_2B_AR antagonism on kidney function and histopathology in diabetic rats. Diabetes was induced in seven-week-old rats by the administration of streptozotocin (STZ) or citrate buffer in control non-diabetic rats. Treatment with the A_2B_AR antagonist MRS1754 (0.5 mg/kg/48 h) was achieved from week 4 after the induction of diabetes in rats. (**A**). The graph depicts the evolution of the proteinuria/creatinine ratio in non-diabetic control rats (green), vehicle-treated diabetic rats (red), and MRS1754-treated diabetic rats (blue). Means ± SEM of samples from 6 control, 6 diabetic, and 6 treated diabetic rats. (**B**). The graph shows FP width from morphometric analyses of 8 transmission electron microscopy images in each rat groups using ImageJ 1.5 software. Images from 2 controls, 2 diabetic, and 3 treated diabetic rats were analyzed. *, *p* < 0.05. (**C**). Representative transmission electron microscopy images of the disposition of the glomerular filtration barrier in control, diabetics, and MRS1754-treated diabetic rats. Arrows indicate FPs. Bar indicates 2 μm.

The functional and structural progressive deterioration of the glomerular filtration barrier was evaluated in STZ-treated rats, a diabetic model resulting from the depletion of pancreatic beta cells. Previously, we have determined that incipient alterations in the diabetic kidney are present following one month from diabetes induction, such as increased glomerular VEGF, TGF-β, and α-SMA levels. Furthermore, an increase in adenosine is triggered at this stage, therefore justifying the pharmacological antagonism of A_2B_AR to evaluate protection on progressive clinical and histopathological signs of DN in diabetic rats [27,30,31,32,33]. In STZ-induced diabetic rats, there is a gradual rise in urinary protein loss over a six-week period post-diabetes induction, with a marked elevation compared to control rats (Figure 1A). A significant outcome generated by A_2B_AR antagonism in diabetic rats was its preventive effect on proteinuria (Figure 1A), which led us to investigate into how the integrity of the glomerular filtration barrier may be affected by diabetes and adenosine signaling intervention. Kidney sections were analyzed by electron microscopy to evaluate the organization and morphology of the FPs. As shown in Figure 1C, the control rats exhibited a stereotypical organization of the filtration barrier, where FPs are regularly interdigitated and have an average foot width of 385 ± 15 nm (Figure 1B). In the diabetic group, there is abnormal cellular morphology; the FPs of the podocytes are effaced, retracted, and broader, reaching a mean width of 585 ± 92 nm, and wide extensions of cell bodies cover the basal membrane. Also, the slit diaphragms are not observable or replaced by occluded-like intercellular junctions. Meanwhile, in the diabetic group treated with MRS1754 (A_2B_AR antagonist), the FPs appear substantially preserved in shape and width (440 ± 20 nm), and regularly interdigitated and intercellular slit diaphragms can be observed (Figure 1B,C). These observations suggest a negative role of A_2B_AR signaling in diabetes that promotes FP reorganization and the slit diaphragm disappearance leading to the disorganization of the podocyte layer, whose arrangement at the filtration barrier provides the compression of the glomerular basal membrane by contractile forces developed by the actomyosin cytoskeleton of the podocytes.

**Figure 2:**
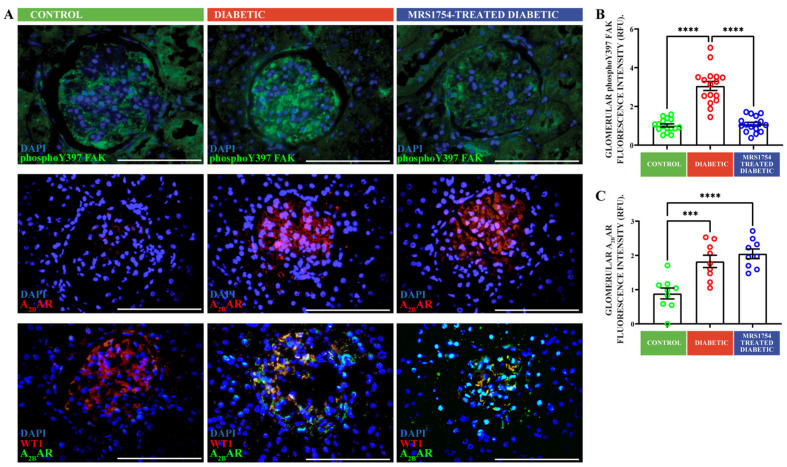
The A_2B_AR antagonism attenuates diabetes-stimulated FAK activation. (**A**). Upper and middle panels show the representative immunofluorescent detection of Y397-phosphorylated focal adhesion kinase (phosphoY397 FAK) and A_2B_AR in kidney sections from control, diabetic, and MRS1754-treated diabetic rats. Images highlight glomerular expression of the proteins. Bottom panel shows representative colocalization analysis of WT1 (red) and A_2B_AR (green) in glomerular cells of rat groups. Tissue nuclei were staining with DAPI (blue). Bar indicates 100 µm. (**B**,**C**). The graphs represent means ± SEM of quantitative analysis of phosphoY397 FAK and A_2B_AR in glomeruli of rat groups. Images of 16 glomerulus from 2 controls, 2 diabetic, and 3 treated diabetic rats were analyzed. ***, *p* < 0.001; ****. *p* < 0.0001.

The induction of A_2B_AR is a remarkable feature of the diabetic kidney disease of human [22] and the STZ-induced diabetic model in rats, as evidenced through immunohistochemical analysis in kidney glomeruli (Figure 2). This induction may affect the signaling properties and cellular responses and, on the other hand, offers the option of the pharmacological intervention of pathological effects. It has been suggested that podocyte FPE proceeds with focal adhesion disassembly [20]. Focal adhesion kinase (FAK) autophosphorylation at tyrosine 397 is critical to focal adhesion turnover, and thus, we aimed to evaluate the consequences of the A_2B_AR blockade on FAK activation. Through immunohistochemical analysis in the kidney sections of experimental rats, we evidenced increased phosphorylated FAK (pY397-FAK) in the glomeruli of diabetic animals (Figure 2A upper panel). Notably, pY397-FAK levels in the diabetic group receiving the A_2B_AR antagonist MRS1754 were similar to the levels found in healthy rats (Figure 2B). The increased levels of the A_2B_AR in diabetic rats (Figure 2A middle panel and Figure 2C) colocalized with the glomerular cells expressing WT1, which is a podocyte-specific lineage marker (Figure 2A bottom panel). Overall, the protective effect of A_2B_AR antagonism on proteinuria in diabetic rats may be linked to the attenuation of podocyte effacement through a FAK-dependent manner.

### 2.2. A_2B_AR Affects Human Immortalized Podocyte Adhesion Dynamics In Vitro

**Figure 3:**
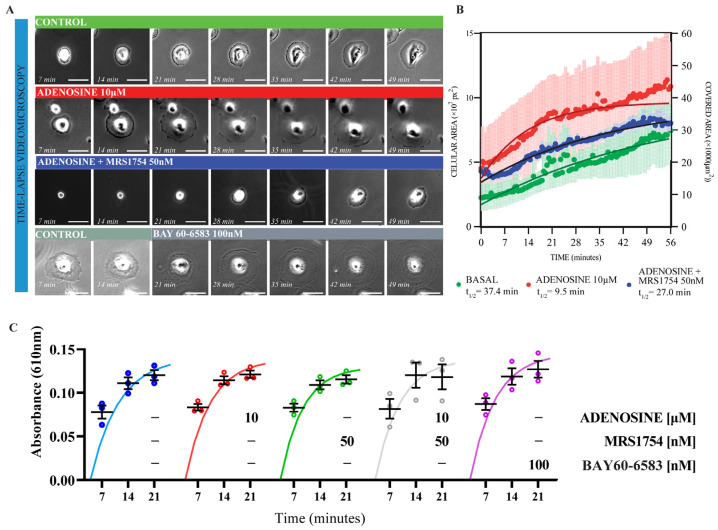
Effects of adenosine and A_2B_AR antagonism on podocytes spreading. (**A**). Representative images of time-lapse video microscopy of cells spreading. Human immortalized podocytes were pre-treated for 24 h with adenosine, antagonist MRS1754, or the combination thereof, and seeded on laminin-coated plates. The effect of the A_2B_AR agonist BAY60-6583 was also evaluated. Bar indicates 100 µm. (**B**). The graph depicts podocytes spreading kinetics from cell treatments in A. The spreading area of a field of view containing approximately 100 individual cells were quantified at 20 s intervals. The average in each point was adjusted to simple association kinetics equation. The graph shows means ± SEM from three independent biological repeats, the non-lineal adjust and the half time value to reach plateau. One-way ANOVA Turkey’s multiple comparison *p* value < 0.0001. (**C**). Adhesion kinetics of podocytes onto laminin-coated plates. Podocytes were pre-treated for 24 h with adenosine, the A_2B_AR agonist BAY60-6583, and the A_2B_AR antagonist MRS1754. Then, podocytes were seeded at 50,000 cells/cm^2^ in medium containing agonists/antagonists and followed for 21 min. The curves represent the equation for the adjustment to simple association kinetics. A statistical analysis of the sum-of-squares F test showed no differences in the association kinetics. *n* = 3.

The alterations associated with podocyte effacement in vivo may correlate with podocyte cytoskeleton rearrangements affecting matrix adhesion and increased cell motility in vitro [34]. To study the effects of A_2B_AR activation on cellular signaling relevant to the glomerular filtration barrier preservation, we choose the thermosensitive immortalized human podocyte model [35]. To emulate the glomerular basal lamina, we employed laminin-521 as a substrate to improve adherence and added supplements emulating the Yaoita et al. [36] conditions for podocyte phenotype development. After 30 days of culture at 37 °C, we obtained differentiated podocytes, which were plated on laminin-coated glass and recorded during the first hour of spreading (Figure 3A). Differentiated podocytes expressed A_2B_AR distributed perinuclear and at the cell periphery (Supplementary Figure S1). Using the instantaneous change in cellular area as a parameter of spreading velocity, we observed that 10 µM adenosine supplementation enhanced the rate at which the podocytes expanded their body over the laminin-coated glass, exhibiting a half-time of expansion of 9 min vs. 37 min in non-stimulated conditions (Figure 3B). Furthermore, the A_2B_AR selective antagonist MRS1754 attenuated the adenosine effect on expansion, resetting the half-time to 27 min (Figure 3B), which highlighted the involvement of the A_2B_AR subtype in the spreading capacity. The kinetics of initial adherence to laminin showed that there is no modification of the initial process of surface receptor engagement. Despite adenosine receptor stimulation (BAY60-6583) or inhibition (MRS1754), the anchorage kinetics were similar to those in untreated cells reaching similar plateau regardless of stimulation or inhibition (Figure 3C). Thus, we hypothesized that the adenosine-stimulated cell body expansion rate was increased mainly through A_2B_AR-mediated cytoskeletal remodeling.

**Figure 4:**
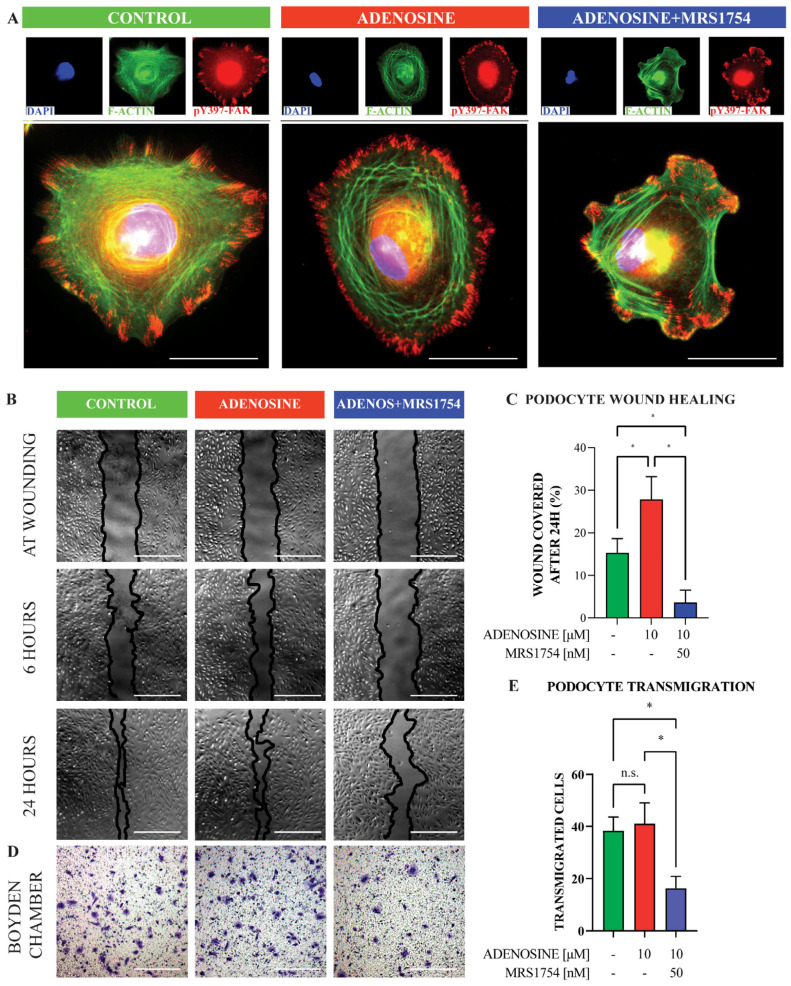
Modulation of A_2B_AR affects the adhesion and migration of podocytes. (**A**). Representative indirect immunofluorescence images of podocytes at a spreading of 60 min and treatments using adenosine (10 μM) or a combination of the A_2B_AR antagonist MRS1754 (50 nM). The phalloidin staining of actin filaments is pseudo colored in green; Y397-phosphorylated FAK is in red; nuclear staining with DAPI is in blue. *n* = 3. Bar indicates 100 µm. (**B**). Representative wound healing assay images. Monolayer cultures of differentiated podocytes were exposed to adenosine and in combination with the A_2B_AR antagonist MRS1754. Light microscopy images were captured at wounding and following 6 and 24 h. Bar indicates 300 µm. (**C**). Quantification of wound healing percentage from images of treated podocytes in (**B**). The graph represents means ± SEM. Five fields were analyzed by each condition. Four independent experiments were achieved. (**D**). Representative images of podocyte transmigration tests through Boyden’s chambers following treatments with adenosine and in combination with the A_2B_AR antagonist MRS1754. Bar indicates 300 µm. (**E**). Quantitative analysis of transmigrated cells from experiments in (**D**). The graph depicts means ± SEM of the number of cells per field, 5 fields analyzed in each condition. *n* = 3. *p* values are shown on brackets in graphs (**C**,**D**) obtained from ANOVA with Fisher’s least significant difference (LSD) post hoc test. *, *p* < 0.05; n.s., no statistical difference.

In fact, there was an evident actin ring enlargement in adenosine-stimulated podocytes compared with control cells (Figure 4A). This actin cytoskeletal arrangement was accompanied by increased pY397-FAK, uniformly located at focal contacts at the cell edge. On the other hand, the MRS1754-treated podocytes lacked uniformity in the pattern of pY397-FAK-enriched adhesions, but focal adhesions appeared to be connected by thick actin stress fibers, which resemble the arrangement observed in control cells (Figure 4A). The stimulation of cells with adenosine enhanced the migratory capability of podocytes both in wound closure experiments (Figure 4B,C) and Boyden chamber transmigration assays (Figure 4D,E), while A_2B_AR antagonism using MRS1754 attenuated the migration ability. These observations may reflect reduced Y397-FAK phosphorylation with A_2B_AR antagonism and could be explained by the decreased tension in the actomyosin system, generating less inside-out activation of FAK and reduced cytoskeletal remodeling.

### 2.3. A_2B_AR Stimulation Increases the Abundance of S19-Phosphorylated Myosin2A

**Figure 5:**
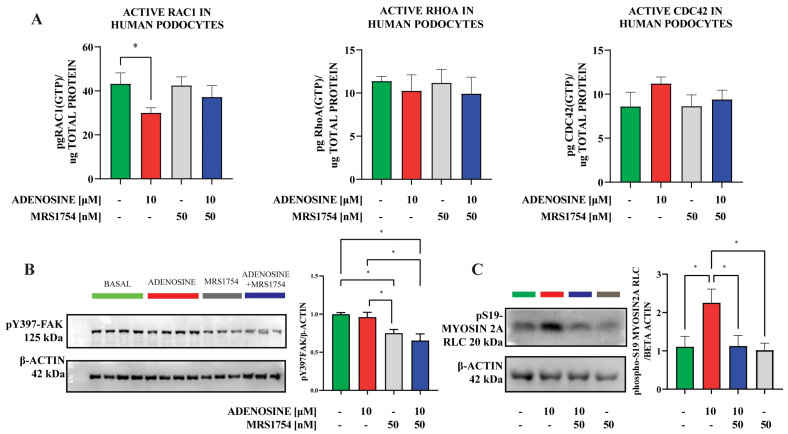
Linking A_2B_AR signaling to cytoskeleton remodeling mediators. (**A**). Quantitative analysis of activated GTP-linked Rho GTPasas (Rac1, RhoA, and CDC42) through G-LISA in podocytes spreading on laminin for 40 min, exposed to adenosine (10 μM), the A_2B_AR antagonist MRS1754 (50 nM), or the combination thereof. The graph depicts the means ± SEM of 4 independent experiments. (**B**). Analysis in Western blots of the relative abundance of Y397-phosphorylated focal adhesion kinase (pY397-FAK) following 40 min of podocytes exposition to adenosine, the A_2B_AR antagonist MRS1754, or the combination thereof. The graph depicts means ± SEM of densitometric analysis of pY397-FAK normalized to β-actin. *n* = 4, basal and adenosine-treated; *n* = 3, MRS1754 and adenosine with MRS1754. (**C**). Analysis in Western blots of the relative abundance of S19-phosphorylated Myosin2A following 40 min of podocytes exposition to adenosine, the A_2B_AR antagonist MRS1754, or the combination thereof. The graph depicts the means ± SEM of densitometric analysis of phosphorylated Myosin2A normalized to β-actin. *n* = 3. *, indicates *p* values < 0.05 between conditions shown in brackets.

Changes in the actin ring appearance and imbalance from a uniform to a discrete pattern of edge adhesion observed when blocking A_2B_AR could implicate the involvement of this receptor in controlling Rho-GTPase activity or actomyosin contractility in podocytes. Using a G-LISA assay, we assessed the activity of the Rho-GTPase system in podocytes treated with adenosine and MRS1754. Our results showed a decrease in the abundance of GTP-loaded Rac1 and slight perturbations to CDC42 in adenosine-stimulated podocytes (Figure 5A). The antagonist MRS1754 returned adenosine-mediated decreased levels to basal conditions (Figure 5A). However, no significant effect could be detected on relative RhoA-GTP levels in any condition (Figure 5A). In contrast, A_2B_AR antagonism had a significant inhibitory effect on FAK activation. Under basal conditions, the levels of FAK activation are maximal because of the spreading stimulus (Figure 5B). As expected, the exposition of cells to adenosine does not increase pY397-FAK over the basal condition. Conversely, the levels of pY397-FAK were reduced in spreading podocytes incubated with MRS1754 compared to basal and adenosine-stimulated conditions (Figure 5B), suggesting that A_2B_AR antagonism negatively influences adhesion remodeling. Previous evidence had shown that the association of FAK to focal adhesions occurs in response to myosin II–mediated cytoskeletal tension, establishing a direct relationship between myosin II phosphorylation and FAK activation [37]. Indeed, we evidenced that the non-muscle Myosin2A, which controls acto-myosin contractility in podocytes, presented increased phosphorylation in the regulatory chain (activating sites threonine18 and serine19) when adenosine was used as a stimulator, but this increased phosphorylation was abrogated when A_2B_AR was blocked by MRS1754 (Figure 5C).

**Figure 6:**
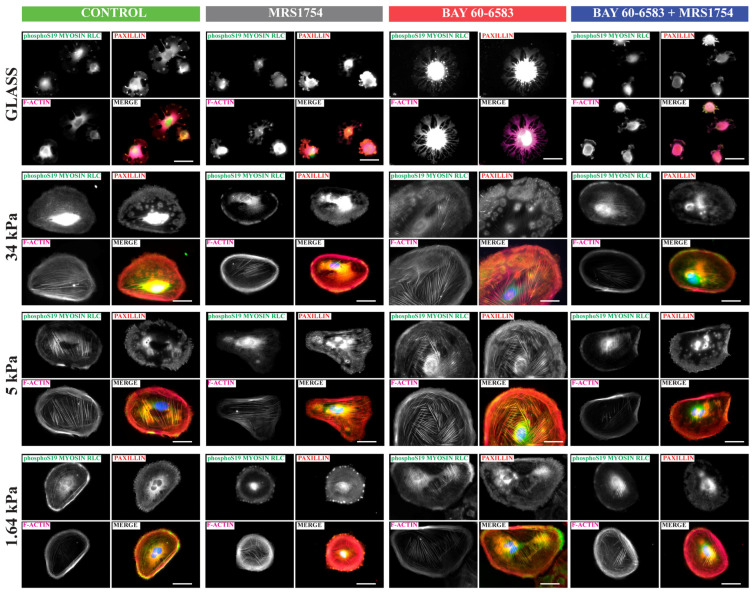
Podocytes display contractility features under A_2B_AR pharmacological modulation. Podocytes were pretreated with A_2B_AR antagonist MRS1754 and the A_2B_AR agonist BAY60-6583. Podocytes were seeded on laminin-coated stiffness-controlled matrices which range from very high (glass) to 34, 5, and 1.64 kPa, containing A_2B_AR pharmacological modulators. After 3 h, the cells were fixed and subjected to the immunofluorescent detection of phopho-Serine18/phospho-Threonine19 Myosin Light chain and Paxillin. Phalloidin and DAPI staining were used for actin filaments and nuclei, respectively. Representative images were captured by epifluorescence microscopy. Bar indicates 50 µm.

We further explored the role of A_2B_AR on phosphorylated Myosin2A in cells expanded in laminin-coated polyacrylamide matrices of controlled stiffness since it allows us to mimic the conditions of in vivo podocytes [38], because cellular contractility in vitro is highly dependent on the stiffness of the substratum used. According to previous measurements, the stiffness of the GBM is in the 2–4 kPa range [39,40]. Furthermore, Embry et al. [41] showed that stiffness in the range of 3–5 kPa enabled the development of structures characteristic of mature podocytes in vivo. Indeed, Figure 6 shows that podocytes on a soft matrix (1.6 kPa) exhibited small focal adhesion complexes with a mostly peripheral location denoted by paxillin staining while in intermediate stiffer conditions (5 kPa and 34 kPa), and the abundance and the adhesion size increased and actin stress fibers appeared co-localized with phosphoS19 Myosin. When cells were seeded on the most rigid matrix (glass), the cells appeared contracted, with thin membrane ruffles and cytoskeletal projections from the cell body resembling pseudopods. Furthermore, the area and abundance of paxillin adhesions was lower than in the intermediate matrices which have more similar stiffness than GBM (Figure 6). When the A_2B_AR was selectively stimulated by the pharmacological agonist BAY60-6583, the cells expanded more broadly and increased the staining of pS19 myosin, which was detected and co-localized with actin cables, suggesting higher contractility potential in intermediate stiffness, while in glass, the pseudopods appeared more prevalent, longer, and highly enriched in peripheral pS19 myosin. This apparent increased contractility potential was not observed when the cells were incubated with MRS1754 alone or in combination with the A_2B_AR agonist, because pS19 myosin positive contractile actin bundles were less prevalent and poorly connected (Figure 6). This effect highlights the plasticity of the podocyte actin cytoskeleton as a function of substrate stiffness, which pulls the adhesion complexes against the forces generated by actomyosin contraction and suggests a role for A_2B_AR stimulation in myosin activation required to activate the molecular clutch in podocytes [42].

## 3. Discussion

The effacement of FPs in podocytes depends on the remodeling of integrin adhesion complexes and the actin cytoskeleton in vivo. This cell behavior bears a resemblance to the motile phenotype of podocytes in vitro [34]. Our results suggest that the chronic increment in adenosine abundance and the induction of the A_2B_AR in diabetic glomeruli play a role in the effacement of FPs, leading to glomerular filtration barrier dysfunction. The use of an A_2B_AR selective antagonist can generate beneficial effects by preserving the arrangement of podocyte FPs and conferring resistance to the development of proteinuria in the diabetic rat model.

The endogenous production of adenosine controls several physiological processes in the kidney, such as the tubular-glomerular feedback (TGF) mechanism that controls the glomerular filtration rate of individual nephrons [23]. An increased flow of filtrate increases sodium abundance in the tubules, and sodium/potassium/chloride NKCC2 co-transporters increase sodium concentration inside macula densa cells [43]. In turn, this triggers the release of ATP, which hydrolyzes to adenosine in the capillary lumen and engages the A1 adenosine receptor for the contraction of the smooth muscle surrounding the afferent arteriole [44]. Thus, adenosine is recognized as a potent vasoconstrictor of afferent arterioles. Its presence in the proximity of glomeruli is expected, but the response exerted to the other cell types inside the glomerulus is not completely understood [45]. The progression of diabetes has been linked with the pathological override of the TGF mechanism by hyperglycemia [46], transferring the whole systemic pressure to the glomeruli. This uncontrolled transcapillary pressure maintains a high intraglomerular strain, progressively challenging the integrity of the GFB. Additionally, hyperglycemia and deficient insulin signaling at the glomerular compartment leads to deficient nucleoside uptake by the cells, consequently leading to chronically elevated extracellular adenosine levels [28,29]. Thus, we propose that adenosine signaling in the diabetic kidney impacts the focal adhesion dynamics of podocytes through the low-affinity A_2B_AR, which negatively affects the glomerular filtration barrier in addition to the rearrangement of the cytoskeleton that weakens the structural integrity required to withstand the capillary pressure. This model is supported by the preservation of the glomerular filtration barrier and the prevention of proteinuria observed in diabetic animals treated with the A_2B_AR antagonist MRS1754. As we showed in this study and as previously evidenced in mice [47], the increased glomerular Y397-phosphorylation of FAK during experimental DN is an event susceptible of intervention to ameliorate podocyte effacement. Previous analyses by Ma et al. [20] demonstrated the critical role of FAK in podocyte effacement since the podocyte-specific deletion of FAK or pharmacologic inactivation of FAK abrogated the proteinuria and FPE in LPS-mediated glomerular injury in mice [20]. Furthermore, the inactivation of FAK demonstrated reduced spreading and migration of podocytes in vitro [20]. Interestingly, in this study, we evidenced that A_2B_AR antagonism was able to attenuate the fraction of pY397-FAK induced by DN in vivo returning to basal levels of pY397-FAK. Also, the antagonism of A_2B_AR reduced a fraction of the spreading-stimulated pY397-FAK in podocytes in vitro. Thus, we propose that the intervened FAK fraction by A_2B_AR antagonism may represent a particular route that mediates focal adhesion instability in pathological conditions.

It is now clearly established that the signaling regulating the plasticity of the actin cytoskeleton in podocytes is of crucial importance for the proper function and maintenance of the glomerular filtration barrier [12,48,49]. In this regard, much attention has been given to the small GTPases of the Rho family as central orchestrators of actin dynamics. Indeed, the podocyte-specific loss of RhoA, Rac1, or CDC42 results in either excess or defective motility that, in both cases, leads to proteinuria [50]. Therefore, the adequate titration of these GTPases is critical for proper functionality. Our results show a mild reduction in relative Rac1-GTP, 40 min after podocytes were stimulated with adenosine. This effect was blocked by MRS1754, showing that A_2B_AR stimulation interrupts the balance of active GTPases, which may explain the accelerated spreading observed in stimulated podocytes. However, Rac1-GTP loading is usually related to actin polymerization in lamellar structures, and the activity of RhoA negatively influences Rac1. Nevertheless, we could not detect a significant change in RhoA levels in stimulated podocytes, indicating that more data regarding the time course of GTP loading in response to A_2B_AR activation are needed to unveil the mechanism involved in the cytoskeletal rearrangements elicited by A_2B_AR in podocytes. Alternatively, a RhoA-independent mechanism that could explain the observed cytoskeletal rearrangements is the influence of A_2B_AR activation on the Gq protein associated with calcium mobilization from intracellular stores through phospholipase C (PLC), generating IP3 and DAG [51]. IP3 rapidly increases cytosolic calcium and elicits calmodulin (CaM) activity. Myosin light chain kinase (MLCK) has been shown to produce the CaM-dependent phosphorylation of the myosin regulatory light chain (RLC) [52,53]. Simultaneously, DAG stimulates PKC activity, inhibiting the myosin light chain phosphatase (MLCP) and sustaining phosphorylated myosin2A RLC levels [53]. The formal increase in phospho-RLC Myosin2A promotes the development of actomyosin contractility; this transmits cytoskeletal tension and induces force-dependent unfolding in adhesion-adaptor proteins, resulting in the recruitment of additional components and adhesion maturation [54]. Enhanced pS19 myosin RLC levels were observed in podocytes when A_2B_AR was stimulated, and it was enriched in branched structures protruding from the cortical actin ring. However, the A_2B_AR/Gq/PLC/IP3/CaM/MLCK/pMyosin or A_2B_AR/Gq/PLC/DAG/PKC/MLCP/pMyosin axis remain to be validated in podocytes. The decreased level of phosphorylated pY397-FAK in podocytes treated with an A_2B_AR antagonist can also be explained by decreased actomyosin tension since FAK recruitment to adhesion sites is strongly dependent on the contractility exerted by myosin2 activity [37].

The increased extracellular adenosine levels and the induction of A_2B_AR turn its signaling properties from an endogenous molecule contributing to renal physiology into a pathogenic factor in DN [22]. Therefore, the dysregulation of this signaling axis is of therapeutic interest. Besides the detrimental effects directly on podocytes, animal models of DN have implicated A_2B_AR in intraglomerular monocyte/macrophage infiltration, polarization, and myofibroblastic transition [31,32,33], involving this receptor subtype in the inflammatory cascade that characterizes DN. Indeed, the cytokine TNF-α, which serves as a crucial transcriptional regulator for the components of the NLRP3 inflammasome [55,56,57], has also been related to A_2B_AR induction in animal models [58]. Interestingly, in patients with DN, the levels of serum TNF-α and its receptor are elevated and are indicative of both renal functional decline and progression to end-stage renal disease [59,60], and inhibiting TNF-α notably alleviated glomerular lesions in murine models of DN [61]. Interception of the inflammatory cascade in DN has also been achieved by the modulation of other purinergic receptors, such as the use of A_2A_ receptor agonist [24] and A_3_ receptor antagonists [62] which denote the susceptibility of renal cells to proinflammatory factors. However, common pathways of anti-inflammatory mechanisms among these interventions remain to be determined. One possible pathway may be that triggered by IL-6, which leads to the phosphorylation of the myosin light chain and focal adhesion instability in podocytes [63].

We conclude that A_2B_AR mediates detrimental effects at the glomerular level in DN, such as the involvement in adhesion dynamics and contractile actin bundle formation that leads to podocyte FPE. Linked to inflammatory cascade and podocyte dysfunction, the blockade of A_2B_AR may represent a novel therapeutic alternative to alleviate glomerular barrier deterioration and the progression of proteinuria.

## 4. Materials and Methods

### 4.1. Experimental Diabetes

Streptozotocin-induced diabetic rats were handled as described by Torres et al. [31]. Briefly, 4-week-old male Sprague Dawley rats were given a single intravenous injection of 65 mg/kg streptozotocin or citrate buffer (10 mM sodium citrate, 0.8% sodium chloride, pH 4.5) as vehicle. Rats with fasting (8 h) blood glucose levels higher than 450 mg/dL were included in the study. Fasting blood glucose levels were monitored in experimental rat groups using a glucometer device (Caresens S, i-Sens Inc., Seoul, Korea). After 4 weeks of diabetes, a group was started on treatment with intraperitoneal injections of the A_2B_AR selective antagonist MRS1754 at 0.5 mg/kg every 48 h. Weekly measurements of body weight, urine output, and urine protein content were carried out (Supplementary Figure S2). Following 12 weeks after diabetes induction, animals were euthanized using sodium thiopental i.p. 100 mg/kg, and kidney sections were prepared for analyses. All animal procedures were approved by the Institutional Committee on the Use of Live Animals in Research at the University Austral de Chile (Ref. 432/2021).

### 4.2. Urine Analyses

Rat urine was recollected in a metabolic cage for 6 h; then, the urine volume was measured (Supplementary Figure S2). Creatinine (cat. number 1260360, Wiener Lab, Rosario, Argentina) and protein (cat. number 1690007, Wiener Lab, Argentina) contents in urine were quantified using systems for the clinical autoanalyzer CM250 (Wiener Lab, Rosario, Argentina).

### 4.3. Transmission Electron Microscopy

Kidneys were fixed with 1% glutaraldehyde in PBS overnight, then immersed in Osmium tetroxide and dehydrated with 50% ethanol and then uranyl acetate in 70% ethanol and finally acetone. Dehydrated sections were embedded in resin and ultrathin sections were cut using a ultramicrotome. Images were analyzed using the ImageJ software, scale was obtained with the calibration bar from TEM, and width was measured in every distinguishable FP. Ten images were taken for every animal, generally containing 10–30 FPs. There were 2 control rats, 2 diabetic rats, and 3 diabetic rats treated with MRS1754 that were used. Data were tabulated and analyzed by ANOVA with (LSD) post hoc test using GraphPad Prism 9.0.

### 4.4. Cell Culture

Human podocyte cell line generated by MA Saleem et al. [35] was transferred for research from the University of Bristol, UK. Podocytes were cultured in polystyrene-treated culture flasks (Nunc Delta Surface). For proliferation, podocytes were cultivated in RPMI medium with 5 mM glucose, 10% fetal bovine serum, insulin-transferrin-selenium supplement (ITS), and penicillin/streptomycin, without interferon-gamma, and incubated at 33.0 °C in 5% CO_2_ until reaching 80% confluence. For differentiation, podocytes were seeded at 50,000 cells/cm^2^ on flasks coated with recombinant human Laminin-521 1 μg per 25 cm^2^ (cat. number A29249, Thermo Fisher Scientific, Waltham, MA, USA) and cultured in RPMI medium with 5 mM glucose supplemented with 0.5% fetal bovine serum, retinoic acid, vitamin D3, ITS, and penicillin/streptomycin in an incubator set to 37 °C, 5% CO_2_. Media were replaced every 48–72 h during a period of one month when pre-treatments and experiments were performed [35].

### 4.5. Time-Lapse Video Microscopy

Differential interference contrast recordings of human immortalized podocytes during spreading on laminin-coated glass were taken using an inverted Zeiss Axio Observer D1 equipped with an incubation stage maintained at 37 °C and 5% CO_2_. Human immortalized podocytes differentiated for 30 days were pre-treated with adenosine receptor agonists/antagonists as indicated before being resuspended by a quick rinse with PBS and 1 min incubation with 500 uL 0.25% trypsin/EDTA at 37 °C. Then, they were softly pipetted up and down 3 times, transferred to 1.5 mL Eppendorf tube, and sedimented for 10 s at 800× *g* in an equilibrated microfuge. The supernatant was carefully aspirated and the pellet was disintegrated into individual cells by three very gentle flicks at the bottom of the tube and resuspended by slowly pipetting up and down 3 times with 200 uL serum free RPMI on ice. Cells were counted using a Neubauer hemocytometer and seeded at a density of 50,000 cells/cm^2^ over a laminin-coated 25 mm coverglass situated inside the imaging chamber containing serum-free RPMI supplemented with adenosine receptor agonists/antagonists as indicated, left to adhere unperturbed during 5 min before recording the spreading for 60 min. Illumination with visible light was set to the minimum intensity necessary to obtain 0.5% saturated pixels at 300 ms capture with CCD camera every 10 s. Images were analyzed with ImageJ software, background subtraction and thresholding processing were made to measure area at every frame. The dynamics of cell spreading follow a universal power law behavior [64,65]. Since we started the recordings with the cells in the disaggregated round form and followed the spreading process until the maximum area of contact was reached, the simple association equation was the best fitting alternative which could enable the comparison of the adhesion kinetics. Data were averaged and adjusted to one-phase association curves Y = Y0 + (Plateau-Y0) (1-exp(-Kx)) with GraphPad Prism 9.0.

### 4.6. Immunofluorescence and Histofluorescence

Cells adhered to coverslips were rinsed with PBS1X, fixed with 4% paraformaldehyde during 15 min at 25 °C, and rinsed and permeabilized 2 min with 0.25% triton X-100 in PBS1X. Then, cells were rinsed and blocked 30 min with blocking solution (3% bovine serum albumin in PBS1X) and then probed with the antibodies anti-paxillin (dilution 1:500, cat. number 05–417, Sigma-Aldrich, St. Louis, MO, USA), anti-actin (dilution 1:1000, cat. CY-SC001, Cytoskeleton Inc., Denver, CO, USA), anti-phospho myosin light chain 2 (Ser19) (dilution 1:1000, cat. number 3671, Cell Signaling Technology, Boston, MA, USA), and anti-phospho FAK (Tyr397) (dilution 1:1000, cat. 31H5L17, Invitrogene, San Diego, CA, USA) in blocking solution during 60 min at 37 °C. After three washes with PBS1X, Alexa Fluor-conjugated secondary antibodies were probed in blocking solution during 60 min at 37 °C, and washed 3 more times before mounting. Tissue immunohistofluorescence was performed in 5 um thin paraffin-embedded kidney sections, deparaffinized with xylene and hydrated by decreasing concentrations of ethanol; then, antigen retrieval was carried out using a citrate buffer (10 mM sodium citrate, 0.05% Tween 20, pH 6.0) by heating it in a slow cooker for 30 min, before being cooled and blocked with 2.5% normal horse serum and 1% bovine serum albumin. Then, samples were incubated overnight with antibody against FAK-phosphorylated on tyrosine 397 (dilution 1:100, cat. number 700255, Invitrogen, San Diego, CA, USA), anti-A_2B_AR (dilution 1:250, cat. orb234999, Biorbyt Ltd., Cambridge, UK), and anti-WT1 (dilution 1:100, cat. sc-393498, Santa Cruz Biotech, Dallas, TX, USA) overnight to 4 °C. Then, the samples were washed and incubated with secondary antibodies Alexa 488 or 568 (dilutions 1:5000, Thermo Fisher Scientific, Waltham, MA, USA). Images of epifluorescence were taken in an inverted Zeiss Axio Observer D1 and analyzed with ImageJ software.

### 4.7. Cell Adhesion Assays

For measuring the cell adhesive capacity, determined by the abundance of surface integrins, human-immortalized podocytes pre-treated with agonist/antagonists of the adenosine receptors indicated were seeded at density of 50,000 cell/cm^2^ in 24-well plated coated with laminin-521, and give them short periods of time (7, 14, and 21 min) incubated at 37 °C, 5% CO_2_, before fixation with PFA 4% in PBS1X and posterior staining with solution 0.1% cristal violet, 20% methanol in PBS1X during 1 h and washed. Then, the stain retained by the adhered podocytes was recovered with 200 uL of methanol and the absorbance at 610 nm was measured in a multi-well spectrophotometer. Results from three independent experiments were plotted and fitted to a one-phase association curve as shown in the graph.

### 4.8. G-LISA

For the quantitative measurement of relative abundance of GTP-loaded Rho GTPases the G-LISA Activation Assay (Cytoskeleton Inc., Denver, CO, USA) was performed as per manufacturer instructions. Cdc42 (BK127-S), Rac1 (BK128-S), and RhoA (BK124-S) were assessed with the respective kits in lysates of human podocytes treated 45 min with adenosine and MRS1754. Four independent experiments were undertaken for each GTPase and data are expressed as individual scatter dots.

### 4.9. Adhesion to Stiffness-Controlled Matrices

For the analysis of podocyte response to adhesion stiffness, we used polyacrylamide gels of controlled stiffness as previously described [38]. Briefly, 12 mm glass coverslips were treated with 3-aminopropyl triethoxysylane, washed and dried, then activated with 0.5% glutaraldehyde in PBS1X before being polymerizing on top of a thin layer of HEPES-buffered polyacrylamide gel at different concentrations to obtain gels of the desired stiffness. Once jellified the surface of the polyacrylamide was coated with 10 uL of 0.1 mg/mL laminin-521 and crosslinking was achieved with 0.1 mg/mL sulpho-SANPAH under UV light. Differentiated podocytes were seeded over the gel at density of 50,000 cells/cm^2^ during 3 h before fixing and proceed to immunofluorescent staining.

### 4.10. Western Blots Analysis

Total proteins were extracted with lysis buffer (2% SDS, 10% glycerol, 63.5 mM Tris HCl, pH 6.8) containing Complete Proteinase Inhibitor (Roche Diagnostics GmbH, Vienna, Austria), 1 µg/mL pepstatin (Roche Diagnostics GmbH, Vienna, Austria) and PhosSTOP^TM^ phosphatase inhibitor (Roche Diagnostics GmbH, Vienna, Austria). The proteins were quantified using BCA protein assay kit (Thermo Fisher Scientific, Waltham, MA, USA). Protein extracts (50 µg) were fractionated by 10% SDS-PAGE and transferred to PVDF membranes. The blots were washed with wash buffer (PBS1x, 0.05% Twheen20), blocked for 1 h with 0.1% BSA, and incubated with primary antibodies anti phospho-myosin light chain 2 (Ser19) (dilution 1:1000, cat. No. 3671, Cell Signaling Technology, Boston, MA, USA), anti phosphoY397-FAK (dilution 1:1000, cat. No. 32835, Cell Signaling Technology, Boston, MA, USA), and anti β-actin (dilution 1:1000, cat. sc-47778, Santa Cruz Biotech, Dallas, TX, USA). The membranes were washed, and the primary antibodies were detected using HRP-coupled secondary antibodies. A chemiluminescence procedure was used for the detection of proteins (Thermo Scientific, Waltham, MA, USA).

### 4.11. Statistical Analysis

Data are expressed as mean ± SEM. Scatter points represent the individual mean of independent experiments and were used for statistical significance tests using GraphPad Prism 9.0 software. All grouped data were analyzed by ANOVA with least significance difference (LSD) post hoc test and *p* values < 0.05 were considered statistically different.

## Data Availability

The raw data supporting the conclusions of this article will be made available by the authors on request.

## Supplementary Materials

The following supporting information can be downloaded at: https://www.mdpi.com/article/10.3390/cells13100846/s1, Figure S1: Indirect immunofluorescence in human immortalized podocytes for the detection of A_2B_AR. Podocytes differentiated 30 days were seeded on laminin-coated coverslips, allowed to spread during 40 min, fixed and subjected to immunostaining as indicated in the methods section. The distribution pattern of A_2B_AR appears mostly perinuclear and at the cell’s periphery in lammelipodium. Representative images were captured by epifluorescence microscopy. Figure S2: Physiological parameters of rats used in the study.

## Abbreviations

A_2B_AR	Adenosine A_2B_ receptor subtype
CKD	Chronic kidney disease
DN	Diabetic nephropathy
FPs	Podocytes foot processes
FPE	Foot processes effacement
GBM	Glomerular basement membrane
GFB	Glomerular filtration barrier
STZ	Streptozotocin
FAK	Focal adhesion kinase

## References

[1] Mills K.T., Xu Y., Zhang W., Bundy J.D., Chen C.S., Kelly T.N., Chen J., He J. (2015). A systematic analysis of worldwide population-based data on the global burden of chronic kidney disease in 2010. Kidney Int..

[2] Webster A.C., Nagler E.V., Morton R.L., Masson P. (2017). Chronic Kidney Disease. Lancet.

[3] Zhang X., Lerman L.O. (2017). The metabolic syndrome and chronic kidney disease. Transl. Res..

[4] Garofalo C., Borrelli S., Minutolo R., Chiodini P., De Nicola L., Conte G. (2017). A systematic review and meta-analysis suggests obesity predicts onset of chronic kidney disease in the general population. Kidney Int..

[5] Sun H., Saeedi P., Karuranga S., Pinkepank M., Ogurtsova K., Duncan B.B., Stein C., Basit A., Chan J.C.N., Mbanya J.C. (2022). IDF Diabetes Atlas: Global, regional and country-level diabetes prevalence estimates for 2021 and projections for 2045. Diabetes Res. Clin. Pract..

[6] Afkarian M., Sachs M.C., Kestenbaum B., Hirsch I.B., Tuttle K.R., Himmelfarb J., de Boer I.H. (2013). Kidney disease and increased mortality risk in type 2 diabetes. J. Am. Soc. Nephrol..

[7] Sun Y.M., Su Y., Li J., Wang L.F. (2013). Recent advances in understanding the biochemical and molecular mechanism of diabetic nephropathy. Biochem. Biophys. Res. Commun..

[8] Jha V., Garcia-Garcia G., Iseki K., Li Z., Naicker S., Plattner B., Saran R., Wang A.Y., Yang C.W. (2013). Chronic kidney disease: Global dimension and perspectives. Lancet.

[9] Kakio Y., Uchida H.A., Takeuchi H., Okuyama Y., Okuyama M., Umebayashi R., Wada K., Sugiyama H., Sugimoto K., Rakugi H. (2018). Diabetic nephropathy is associated with frailty in patients with chronic hemodialysis. Geriatr. Gerontol. Int..

[10] Pavenstädt H., Kriz W., Kretzler M. (2003). Cell biology of the glomerular podocyte. Physiol. Rev..

[11] Turing A.M. (1990). The chemical basis of morphogenesis. Bull Math Biol..

[12] Faul C., Asanuma K., Yanagida-Asanuma E., Kim K., Mundel P. (2007). Actin up: Regulation of podocyte structure and function by components of the actin cytoskeleton. Trends Cell Biol..

[13] Kriz W., Lemley K.V. (2017). Mechanical challenges to the glomerular filtration barrier: Adaptations and pathway to sclerosis. Pediatr. Nephrol..

[14] Kriz W., Shirato I., Nagata M., LeHir M., Lemley K.V. (2013). The podocyte’s response to stress: The enigma of foot process effacement. Am. J. Physiol. Renal Physiol..

[15] Pan Y., Jiang S., Hou Q., Qiu D., Shi J., Wang L., Chen Z., Zhang M., Duan A., Qin W. (2018). Dissection of Glomerular Transcriptional Profile in Patients with Diabetic Nephropathy: SRGAP2a Protects Podocyte Structure and Function. Diabetes.

[16] Gojo A., Utsunomiya K., Taniguchi K., Yokota T., Ishizawa S., Kanazawa Y., Kurata H., Tajima N. (2007). The Rho-kinase inhibitor, fasudil, attenuates diabetic nephropathy in streptozotocin-induced diabetic rats. Eur. J. Pharmacol..

[17] Komers R., Oyama T.T., Beard D.R., Tikellis C., Xu B., Lotspeich D.F., Anderson S. (2011). Rho kinase inhibition protects kidneys from diabetic nephropathy without reducing blood pressure. Kidney Int..

[18] Wang W., Wang Y., Long J., Wang J., Haudek S.B., Overbeek P., Chang B.H., Schumacker P.T., Danesh F.R. (2012). Mitochondrial fission triggered by hyperglycemia is mediated by ROCK1 activation in podocytes and endothelial cells. Cell Metab..

[19] Kumagai T., Baldwin C., Aoudjit L., Nezvitsky L., Robins R., Jiang R., Takano T. (2014). Protein tyrosine phosphatase 1B inhibition protects against podocyte injury and proteinuria. Am. J. Pathol..

[20] Ma H., Togawa A., Soda K., Zhang J., Lee S., Ma M., Yu Z., Ardito T., Czyzyk J., Diggs L. (2010). Inhibition of podocyte FAK protects against proteinuria and foot process effacement. J. Am. Soc. Nephrol..

[21] Sever S., Schiffer M. (2018). Actin dynamics at focal adhesions: A common endpoint and putative therapeutic target for proteinuric kidney diseases. Kidney Int..

[22] Oyarzún C., Garrido W., Alarcón S., Yáñez A., Sobrevia L., Quezada C., San Martín R. (2017). Adenosine contribution to normal renal physiology and chronic kidney disease. Mol. Asp. Med..

[23] Schnermann J., Levine D.Z. (2003). Paracrine factors in tubuloglomerular feedback: Adenosine, ATP, and nitric oxide. Annu. Rev. Physiol..

[24] Awad A.S., Rouse M., Liu L., Vergis A.L., Rosin D.L., Linden J., Sedor J.R., Okusa M.D. (2008). Activation of adenosine 2A receptors preserves structure and function of podocytes. J. Am. Soc. Nephrol..

[25] Xia J.F., Liang Q.L., Hu P., Wang Y.M., Li P., Luo G.A. (2009). Correlations of six related purine metabolites and diabetic nephropathy in Chinese type 2 diabetic patients. Clin. Biochem..

[26] Pak E.S., Cha J.J., Cha D.R., Kanasaki K., Ha H. (2022). Adenosine receptors as emerging therapeutic targets for diabetic kidney disease. Kidney Res. Clin. Pract..

[27] Kretschmar C., Oyarzún C., Villablanca C., Jaramillo C., Alarcón S., Perez G., Díaz-Encarnación M.M., Pastor-Anglada M., Garrido W., Quezada C. (2016). Reduced Adenosine Uptake and Its Contribution to Signaling that Mediates Profibrotic Activation in Renal Tubular Epithelial Cells: Implication in Diabetic Nephropathy. PLoS ONE.

[28] Alarcón S., Garrido W., Vega G., Cappelli C., Suárez R., Oyarzún C., Quezada C., San Martín R. (2017). Deficient Insulin-mediated Upregulation of the Equilibrative Nucleoside Transporter 2 Contributes to Chronically Increased Adenosine in Diabetic Glomerulopathy. Sci. Rep..

[29] Suarez R., Villarreal C., Nahuelpán Y., Jara C., Oyarzún C., Alarcón S., Díaz-Encarnación M.M., Guillén-Gómez E., Quezada C., San Martín R. (2024). Defective insulin-stimulated equilibrative nucleoside transporter-2 activity and altered subcellular transporter distribution drive the loss of adenosine homeostasis in diabetic kidney disease progression. Biochim. Biophys. Acta Mol. Basis Dis..

[30] Roa H., Gajardo C., Troncoso E., Fuentealba V., Escudero C., Yáñez A., Sobrevia L., Pastor-Anglada M., Quezada C., San Martin R. (2009). Adenosine mediates transforming growth factor-beta 1 release in kidney glomeruli of diabetic rats. FEBS Lett..

[31] Torres Á., Muñoz K., Nahuelpán Y., Saez A.P.R., Mendoza P., Jara C., Cappelli C., Suarez R., Oyarzún C., Quezada C. (2020). Intraglomerular Monocyte/Macrophage Infiltration and Macrophage-Myofibroblast Transition during Diabetic Nephropathy Is Regulated by the A_2B_ Adenosine Receptor. Cells.

[32] Cárdenas A., Toledo C., Oyarzún C., Sepúlveda A., Quezada C., Guillén-Gómez E., Díaz-Encarnación M.M., Pastor-Anglada M., San Martín R. (2013). Adenosine A_2B_ receptor-mediated VEGF induction promotes diabetic glomerulopathy. Lab. Invest..

[33] Torres-Arévalo Á., Nahuelpán Y., Muñoz K., Jara C., Cappelli C., Taracha-Wiśniewska A., Quezada-Monrás C., Martín R.S. (2023). A_2B_AR Antagonism Decreases the Glomerular Expression and Secretion of Chemoattractants for Monocytes and the Pro-Fibrotic M2 Macrophages Polarization during Diabetic Nephropathy. Int. J. Mol. Sci..

[34] Reiser J., Sever S. (2013). Podocyte biology and pathogenesis of kidney disease. Annu. Rev. Med..

[35] Saleem M.A., O’Hare M.J., Reiser J., Coward R.J., Inward C.D., Farren T., Xing C.Y., Ni L., Mathieson P.W., Mundel P. (2002). A conditionally immortalized human podocyte cell line demonstrating nephrin and podocin expression. J. Am. Soc. Nephrol..

[36] Yaoita E., Yoshida Y., Nameta M., Takimoto H., Fujinaka H. (2018). Induction of interdigitating cell processes in podocyte culture. Kidney Int..

[37] Pasapera A.M., Schneider I.C., Rericha E., Schlaepfer D.D., Waterman C.M. (2010). Myosin II activity regulates vinculin recruitment to focal adhesions through FAK-mediated paxillin phosphorylation. J. Cell Biol..

[38] Fischer R.S., Myers K.A., Gardel M.L., Waterman C.M. (2012). Stiffness-controlled three-dimensional extracellular matrices for high-resolution imaging of cell behavior. Nat. Protoc..

[39] Levental I., Levental K.R., Klein E.A., Assoian R., Miller R.T., Wells R.G., Janmey P.A. (2010). A simple indentation device for measuring micrometer-scale tissue stiffness. J. Phys. Condens. Matter.

[40] Wyss H.M., Henderson J.M., Byfield F.J., Bruggeman L.A., Ding Y., Huang C., Suh J.H., Franke T., Mele E., Pollak M.R. (2011). Biophysical properties of normal and diseased renal glomeruli. Am. J. Physiol. Cell Physiol..

[41] Embry A.E., Mohammadi H., Niu X., Liu L., Moe B., Miller-Little W.A., Lu C.Y., Bruggeman L.A., McCulloch C.A., Janmey P.A. (2016). Biochemical and Cellular Determinants of Renal Glomerular Elasticity. PLoS ONE.

[42] Elosegui-Artola A., Oria R., Chen Y., Kosmalska A., Pérez-González C., Castro N., Zhu C., Trepat X., Roca-Cusachs P. (2016). Mechanical regulation of a molecular clutch defines force transmission and transduction in response to matrix rigidity. Nat. Cell Biol..

[43] Castrop H. (2007). Mediators of tubuloglomerular feedback regulation of glomerular filtration: ATP and adenosine. Acta Physiol..

[44] Thomson S., Bao D., Deng A., Vallon V. (2000). Adenosine formed by 5′-nucleotidase mediates tubuloglomerular feedback. J. Clin. Investig..

[45] Peti-Peterdi J. (2006). Calcium wave of tubuloglomerular feedback. Am. J. Physiol. Renal Physiol..

[46] Thomson S.C., Vallon V., Blantz R.C. (2004). Kidney function in early diabetes: The tubular hypothesis of glomerular filtration. Am. J. Physiol. Renal Physiol..

[47] Guo K., Pan P., Wu M., Ma Y., Lu J., Chen H. (2020). Hyposialylated angiopoietin-like-4 induces apoptosis of podocytes via β1 Integrin/FAK signaling in diabetic nephropathy. Mol. Cell. Endocrinol..

[48] Mundel P., Reiser J. (2010). Proteinuria: An enzymatic disease of the podocyte?. Kidney Int..

[49] Greka A., Mundel P. (2012). Cell biology and pathology of podocytes. Annu. Rev. Physiol..

[50] Matsuda J., Asano-Matsuda K., Kitzler T.M., Takano T. (2021). Rho GTPase regulatory proteins in podocytes. Kidney Int..

[51] Feoktistov I., Biaggioni I. (1995). Adenosine A2b receptors evoke interleukin-8 secretion in human mast cells. An enprofylline-sensitive mechanism with implications for asthma. J. Clin. Investig..

[52] Somlyo A.P., Somlyo A.V. (2000). Signal transduction by G-proteins, rho-kinase and protein phosphatase to smooth muscle and non-muscle myosin II. J. Physiol..

[53] Somlyo A.P., Somlyo A.V. (2003). Ca^2+^ sensitivity of smooth muscle and nonmuscle myosin II: Modulated by G proteins, kinases, and myosin phosphatase. Physiol. Rev..

[54] Parsons J.T., Horwitz A.R., Schwartz M.A. (2010). Cell adhesion: Integrating cytoskeletal dynamics and cellular tension. Nat. Rev. Mol. Cell Biol..

[55] McGeough M.D., Wree A., Inzaugarat M.E., Haimovich A., Johnson C.D., Peña C.A., Goldbach-Mansky R., Broderick L., Feldstein A.E., Hoffman H.M. (2017). TNF regulates transcription of NLRP3 inflammasome components and inflammatory molecules in cryopyrinopathies. J. Clin. Investig..

[56] Amaral F.A., Bastos L.F., Oliveira T.H., Dias A.C., Oliveira V.L., Tavares L.D., Costa V.V., Galvão I., Soriani F.M., Szymkowski D.E. (2016). Transmembrane TNF-α is sufficient for articular inflammation and hypernociception in a mouse model of gout. Eur. J. Immunol..

[57] Cheng D., Liang R., Huang B., Hou J., Yin J., Zhao T., Zhou L., Wu R., Qian Y., Wang F. (2019). Tumor necrosis factor-α blockade ameliorates diabetic nephropathy in rats. Clin. Kidney J..

[58] St Hilaire C., Koupenova M., Carroll S.H., Smith B.D., Ravid K. (2008). TNF-α upregulates the A_2B_ adenosine receptor gene: The role of NAD(P)H oxidase 4. Biochem. Biophys. Res. Commun..

[59] Coca S.G., Nadkarni G.N., Huang Y., Moledina D.G., Rao V., Zhang J., Ferket B., Crowley S.T., Fried L.F., Parikh C.R. (2017). Plasma Biomarkers and Kidney Function Decline in Early and Established Diabetic Kidney Disease. J. Am. Soc. Nephrol..

[60] Niewczas M.A., Gohda T., Skupien J., Smiles A.M., Walker W.H., Rosetti F., Cullere X., Eckfeldt J.H., Doria A., Mayadas T.N. (2012). Circulating TNF receptors 1 and 2 predict ESRD in type 2 diabetes. J. Am. Soc. Nephrol..

[61] Awad A.S., You H., Gao T., Cooper T.K., Nedospasov S.A., Vacher J., Wilkinson P.F., Farrell F.X., Brian Reeves W. (2015). Macrophage-derived tumor necrosis factor-α mediates diabetic renal injury. Kidney Int..

[62] Garrido W., Jara C., Torres A., Suarez R., Cappelli C., Oyarzún C., Quezada C., San Martín R. (2019). Blockade of the Adenosine A_3_ Receptor Attenuates Caspase 1 Activation in Renal Tubule Epithelial Cells and Decreases Interleukins IL-1β and IL-18 in Diabetic Rats. Int. J. Mol. Sci..

[63] He F.F., Bao D., Su H., Wang Y.M., Lei C.T., Zhang C.Y., Ye C., Tang H., Wan C., You C.Q. (2018). IL-6 increases podocyte motility via MLC-mediated focal adhesion impairment and cytoskeleton disassembly. J. Cell. Physiol..

[64] Cuvelier D., Théry M., Chu Y.S., Dufour S., Thiéry J.P., Bornens M., Nassoy P., Mahadevan L. (2007). The universal dynamics of cell spreading. Curr. Biol..

[65] Murrell M., Pontani L.L., Guevorkian K., Cuvelier D., Nassoy P., Sykes C. (2011). Spreading dynamics of biomimetic actin cortices. Biophys. J..

