# Supplementary material for: Pharmacological Blockade of the Adenosine A2B Receptor Is Protective of Proteinuria in Diabetic Rats, through Affecting Focal Adhesion Kinase Activation and the Adhesion Dynamics of Podocytes
Source: Cells. 2024 May 16;13(10):846. doi: 10.3390/cells13100846 (PMC11119713; doi:10.3390/cells13100846)
Supplement: Supplementary file 1 [file cells-13-00846-s001.zip › cells-2920960-supplementary.pdf]

Supplementary Figure S1

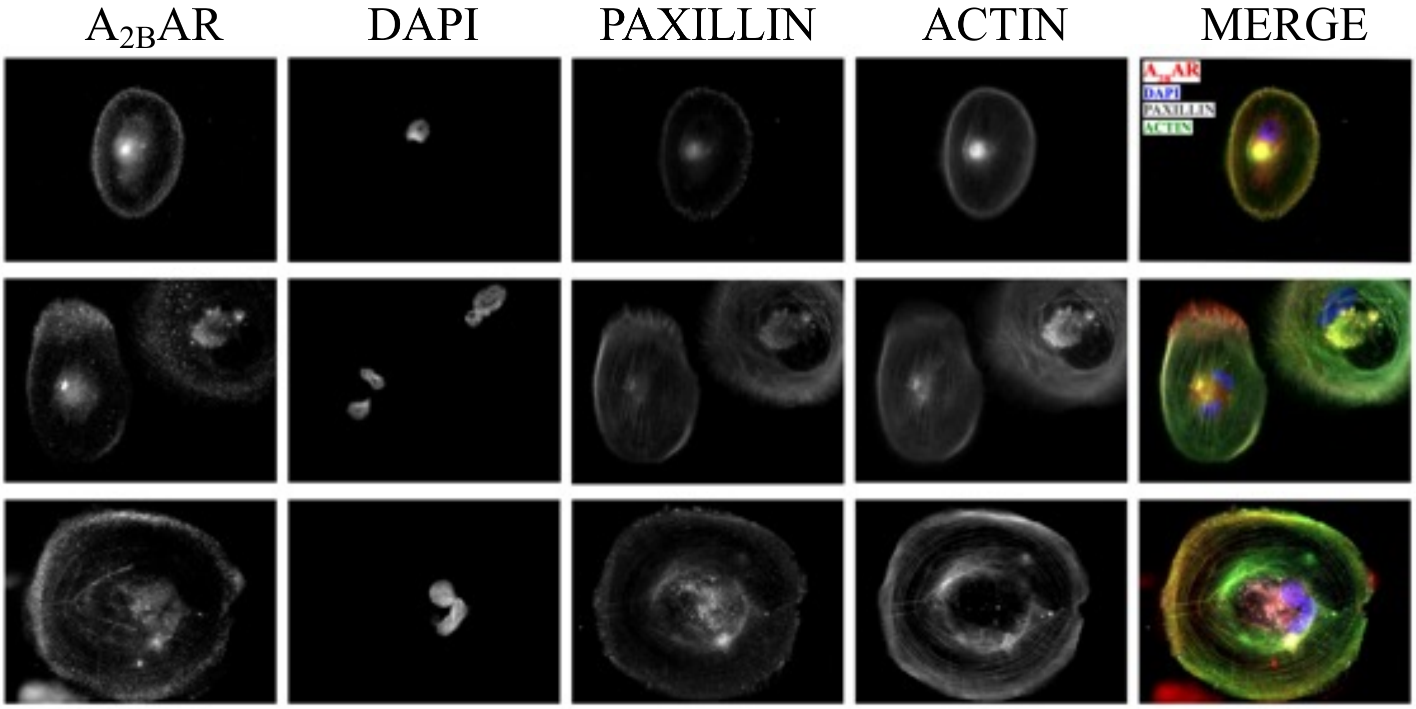

Indirect immunofluorescence in human immortalized podocytes for the detection of $A_{2B}AR$. Podocytes differentiated 30 days were seeded on laminin-coated coverslips, allowed to spread during 40 minutes, fixed and subjected to immunostaining as indicated in the methods section. The distribution pattern of $A_{2B}AR$ appears mostly perinuclear and at the cell's periphery in lammelipodium. Representative images were captured by epifluorescence microscopy.

Supplementary Figure S2

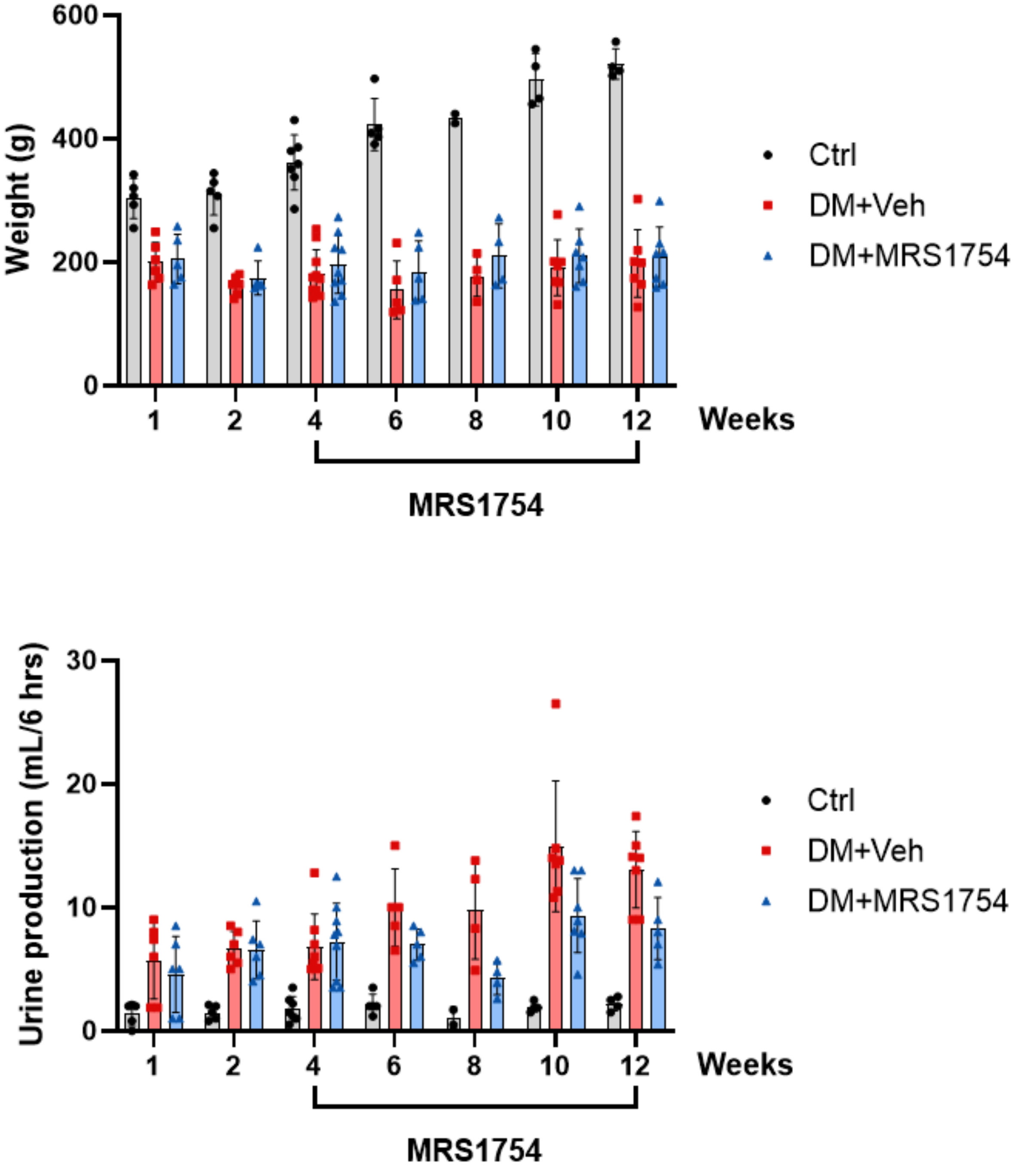

Physiological parameters of rats used in the study.
